# Elementary Chemistry, Theoretical and Practical

**Published:** 1851-07

**Authors:** 


*Elementary Chemistry, Theoretical and Practical.* By George Fownes, F. R. S. &c. Third American from a late London edition, with numerous wood engravings. Philadelphia: Lea & Blanchard, 1850.

It is unnecessary to speak of this reliable, convenient, sizable, cheap, interesting, well known and popular treatise. We are glad to see a third edition.

